# Anxiety and Gastrointestinal Symptoms Related to COVID-19 during Italian Lockdown

**DOI:** 10.3390/jcm10061221

**Published:** 2021-03-16

**Authors:** Ludovico Abenavoli, Pietro Cinaglia, Giuditta Lombardo, Eduardo Boffoli, Miriam Scida, Anna Caterina Procopio, Tiziana Larussa, Luigi Boccuto, Christian Zanza, Yaroslava Longhitano, Sharmila Fagoonee, Francesco Luzza

**Affiliations:** 1 Department of Health Sciences, University “Magna Graecia”, 88100 Catanzaro, Italy; procopioannacaterina@gmail.com (A.C.P.); tarussa@unicz.it (T.L.); luzza@unicz.it (F.L.); 2 Department of Surgical and Medical Sciences, University “Magna Graecia”, 88100 Catanzaro, Italy; cinaglia@unicz.it; 3 Independent Researcher, 88100 Catanzaro, Italy; giuditta.lombardo@libero.it; 4 School of Medicine, University “Magna Graecia”, 88100 Catanzaro, Italy; eduardo.boffoli96@gmail.com (E.B.); scidamiriam0@gmail.com (M.S.); 5 School of Nursing, College of Behavioral, Social and Health Sciences, Clemson University, Clemson, SC 29634, USA; lboccut@clemson.edu; 6 Department of Anesthesia, Critical Care Medicine and Emergency Medicine, Michele and Pietro Ferrero Hospital, 12060 Verduno, Italy; christian.zanza@live.it; 7 Department of Anesthesia and Critical Care Medicine, AON SS Antonio e Biagio e Cesare Arrigo, 15121 Alessandria, Italy; lon.yaro@gmail.com; 8 Institute of Biostructure and Bioimaging, National Research Council, Molecular Biotechnology Center, 10135 Turin, Italy; sharmila.fagoonee@unito.it

**Keywords:** medical students, public health, social media, dietary habit, pandemic

## Abstract

The first case of infection by SARS-CoV-2 (i.e., COVID-19) was officially recorded by the Italian National Health Service on 21 February 2020. Respiratory tract manifestations are the most common symptoms, such as gastrointestinal symptoms (GISs) like nausea or sickness, diarrhea, and anorexia, and psychological effects may be reported in affected individuals. However, similar symptoms may be observed in healthy people as a consequence of an anxiety state. Methods: We analyzed GISs and anxiety state during the COVID-19 lockdown period; from 9 March 2020 to 4 May 2020. A web-based survey consisting of 131 items was administered to 354 students affiliated with the School of Medicine of the University “Magna Graecia” of Catanzaro; Italy. A set of statistical analyses was performed to analyze the relationships among the answers to assess a correlation between the topics of interest. Results: The statistical analysis showed that 54.0% of interviewed reported at least one GISs, 36.16% of which reported a positive history for familial GISs (FGISs). The 354 subjects included in our cohort may be stratified as follows: 25.99% GISs and FGISs, 27.97% GISs and no-FGISs, 10.17% no-GISs and FGISs, 35.87% no-GISs and no-FGISs. Results indicated an anxiety state for 48.9% of respondents, of which 64.74% also presented GISs. In addition, considered dietary habits, we detect the increased consumption of hypercaloric food, sweetened drinks, and alcoholic beverages. Conclusions: The increase of GISs during the lockdown period in a population of medical students, may be correlated to both dietary habits and anxiety state due to a concern for one’s health.

## 1. Introduction

The World Health Organization (WHO) defines Coronavirus Disease 2019 (COVID-19) as a serious challenge to the world public health. On 31 December 2019, a cluster of pneumonia cases of unknown etiology was reported in Wuhan, a Chinese city in the Hubei Province [1]. COVID-19 involves principally respiratory tract, and the clinical presentation was very similar to that recorded during the Severe Acute Respiratory Syndrome (SARS) outbreak of 2003 [2,3]. The virus rapidly spread worldwide, and it is responsible for a pandemic state with relevant clinical, psychological and economic consequences [4,5,6].

In Italy, the first case of infection by SARS Coronavirus 2 (SARS-CoV-2) was officially recorded on 21 February 2020 and on 11 March 2020, the WHO declared the COVID-19 outbreak a global pandemic and the Italian government enforced a lockdown measure, which is defined as a state of isolation and restricted access [7,8]. The lockdown period in Italy extended from 11 March to the following two months exactly until 4 May 2020.

The COVID-19 pandemic has influenced all aspects of human life with an unprecedented global crisis characterized by drastic changes in social life, personal freedom, and economic activities and has created distress, as well as exacerbation of mental health issues in a traumatic stress context, especially for healthcare workers [9].

Literature supports the association between anxiety and gastrointestinal symptoms (GISs) [10,11]. In subjects with functional gastrointestinal disorders, such as dyspepsia and irritable bowel syndrome, the presence of anxiety seems to play a major role in the genesis and the perception of symptoms [12]. The gastrointestinal tract and the nervous system are intimately connected via bidirectional signaling mechanisms characterized by neural, endocrine, and immune pathways, in a context knows as the “gut–brain axis” [13]. In particular, the central stress circuitry is the neural network that receives input from the somatic and visceral afferent systems and also from the visceral motor cortex and generates the stress response [14]. In this way, stress conditions could increase gastrointestinal motility and visceral sensitivity [15].

It has been proven that anxiety can affect many university students, particularly the ones affiliated with medical schools [16]. Indeed, medical students differ from the general population in terms of the high academic and professional standards placed on them. They are under constant stress due to the duration of their study plan characterized by higher work overload with physical and mental exhaustion. Recent studies conducted among medical students clearly report their psychological status concerning the present pandemic [17,18,19].

In this way, our study aimed to analyze GISs before and during the Italian lockdown for SARS-CoV-2 and the possible correlations of the lockdown on both the anxiety state and the referred changes in dietary habits in a population of students affiliated with the School of Medicine at the University “Magna Graecia” (UMG) of Catanzaro, Italy.

## 2. Materials and Methods

### 2.1. Study Design

We designed a web-based survey and eventually administered it to 354 medical students. Data has been acquired by using an online recruitment strategy over a period between 9 March 2020 and 4 May 2020. Our study was performed on 354 medical students, of which 111 males (31.4%) and 243 females (68.6%).

The following exclusion criteria were applied: (i) self-reported positivity to COVID-19, and (ii) subject not affiliated with the School of Medicine of the UMG, Italy. Both have been used to deny access to the survey, therefore, only the subjects who satisfied these criteria were allowed to complete the survey, while the others were discarded and unregistered. The second criterion has been checked limiting the access to the email domain released by UMG in order to check their affiliation. Our survey consists of 131 items, and it was designed as follow:

- 8 questions related to socio-demographic characteristics;
- 17 questions related to past medical history for GISs;
- 2 sets (before and during lockdown, respectively) of 17 questions related to the presence of GISs;
- 2 sets (before and during lockdown, respectively) of 10 questions to evaluate dietary habits;
- 2 sets (before and during lockdown, respectively) of 26 questions for anxiety evaluation, of these 12 indicate the common self-reported symptoms related to anxiety and 14 represent the Short Health Anxiety Inventory (SHAI) test, a psychometrically sound tool for assessing health anxiety [20].

According to SHAI, the related answers are based on a Likert-type scale representing a score defined in a range 0–3 for never, rarely, sometimes, and often, respectively.

It was intended to collect data related to (i) socio-demographic characteristics, (ii) GISs, and (iii) psychometric parameters. An exhaustive representation of our questionnaire has been reported in Table S1 (see Supplementary Materials).

### 2.2. Statistical Analysis

Statistical analyses have been performed both to explore the relationships among the answers and to assess a correlation between the topics of interest over the SARS-CoV-2 lockdown: occurrence of GISs, and the influence of anxiety state on these symptoms. All the computations were carried out by using IBM SPSS Statistics (IBM Corp., Armonk, NY, USA). Each test is chosen according to the type of data representing the information of interest, as appropriate.

Likert-type data may be analyzed by using nonparametric tests such as Spearman’s correlation, and Wilcoxon signed-rank test. Latter represents an optimal solution when independent variables consist of two categorical and related groups that make the application of a classic dependent *t*-test inappropriate. For instance, we use this test to correlate the GISs with the dietary habits within the same period. Otherwise, *t*-test and ANOVA have been used to analyze the changes over time; for instance, to evaluate the changes from pre-lockdown to during-lockdown, we use a Paired-samples *t*-test for scale data (e.g., level anxiety), and ANOVA for ordinal data (e.g., values in Likert-type scale). Each test has been chosen according to the type of data (e.g., ordinal, nominal, scale) to be analyzed and the period, as appropriate [21].

Furthermore, a preliminary assessment has been performed by using a descriptive analysis for summarizing data frequency and tendency. We codified the multiple-choice from raw text into integer values in order to define the related Likert-type scale assigning a progressive score for each answer. An independent-sample *t*-test has been performed on the anxiety level by using GISs as a grouping variable. It reports both the *t*-test for equality of means and Levene’s test for equality of variances. Therefore, the anxiety state has been correlated with the presence of GISs by performing a bivariate correlation and a test of significance for two-tailed. Spearman’s correlation has been used to study the psychometric parameters to evaluate the related anxiety state. For all subjects studied, a mathematical function has been applied in order to calculate the anxiety level by assigning to each answer a score; the final score is the sum of all scores related to psychometric evaluation. According to SHAI, we assumed the existence of an anxiety state for an anxiety level with a final score greater than or equal to 18. The periods before and during SARS-CoV-2 lockdown have been analyzed by performing an ANOVA test, to evaluate also how data changes within groups as changes in dietary habits, while a paired-samples *t*-test. Graphical representation of the figures is depicted by using contingency tables or crosstabs, in order to summarize the relationship between several categorical variables. Contingency tables do not report the statistical significance, as well as the bar chart used to summarize results in the before-during analysis. Therefore, the statistical significance has been evaluated by using the statistical tests described above for each set of data separately. We assume a value as statistically significant for a *p*-value less than 0.05.

### 2.3. Ethical Considerations

The study was conducted in accordance with the Helsinki Declaration. The participants received oral and written information regarding the study. All participants were informed that participation was voluntary and that they could withdraw at any time without consequences. The study protocol was approved by the local Research Ethics Committee (n.221/2020), and informed consent was obtained from all participants.

## 3. Results

Table S2 (see Supplementary Materials) summarizes demographic data related to subjects recruited for this study.

**Figure 1:**
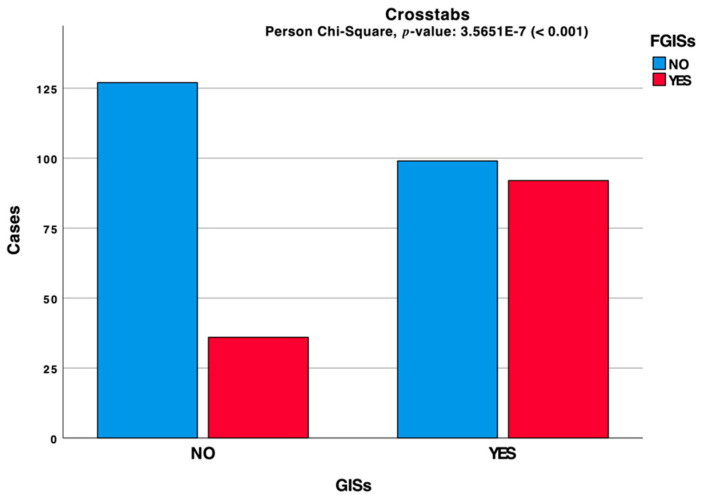
Clustered bar chart based on the contingency table related to the interactions between GISs and FGISs during the lockdown period.

The statistical analysis conducted on our cohort showed that 54.0% of subjects interviewed, self-reported GISs. A clustered bar chart based on the contingency table related to the interactions between these symptoms is depicted in Figure 1 and shows 36.16% of the cohort students having positive history for familial GISs (FGISs). The 354 subjects in our sample may be grouped as follows: 25.99% GISs and FGISs, 27.97% GISs and no-FGDs, 10.17% no-GISs and FGISs, 35.87% no-GISs and no-FGISs. A nonparametric correlation based on the Spearman method has been performed to analyze the relationship between GISs and FGISs. Our results showed a high statistical significance between these parameters.

**Figure 2:**
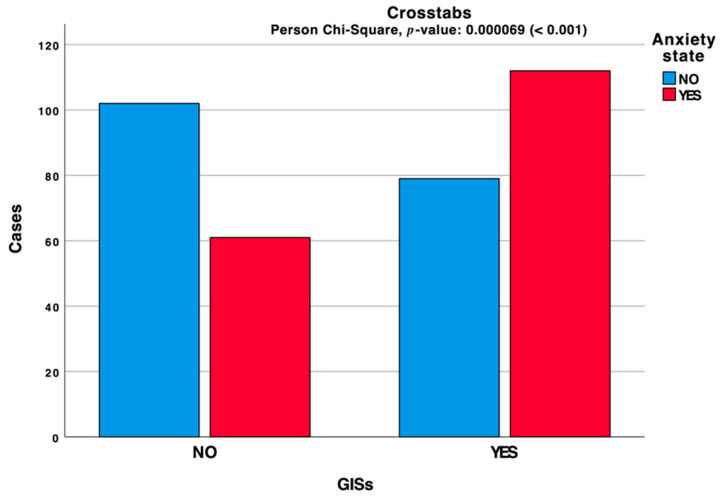
Clustered bar chart based on the contingency table related to the interactions between GISs and anxiety state during the lockdown period.

The collected data confirmed a relationship between GISs and FGISs. A detailed analysis assessed an anxiety state for 48.9% of interviewed, of which 64.74% reported also GISs (Figure 2).

Other analyses have been performed to study the relationships between anxiety level and the presence of GISs, as well as between the latter and the anxiety state. Table 1 reports the frequency analysis for the related anxiety state during the lockdown. Table 2 reports the correlation between this one and the presence of GISs, during the lockdown.

The anxiety level analysis (Table 3) shows that its mean value was 13.85 in pre-lockdown, while it increased to 18.18 during-lockdown (+31.26%); this increase was statistically significant.

Furthermore, it has been correlated with the presence of GISs during the lockdown by performing an independent-sample t-test. Table 4 shows the related group statistics, while Table 5 shows both equality of means and Levene’s test for equality of variances. GISs have been analyzed to study their evolution during the SARS-CoV-2 lockdown period. The information related to the GISs and dietary habits has been evaluated before and during the SARS-CoV-2 lockdown period and correlated by performing a Wilcoxon signed-rank test.

The latter has been structured on two sets of answers, each one concerning the period before and during the lockdown. Table 6 and Table 7 show the resultant output. For each pair of questions has been calculated if the correlation between these ones is statistically significant, thus if it may be used to provide evidence concerning the likelihood of their change during the lockdown period. Table 6 show that correlations between GISs before and during the SARS-CoV-2 lockdown are all statistically significant, while Table 7 show an increase in the consumption of specific food as meat, pizza, pre-cooked food, alcohol beverages, and sweetened drinks.

**Figure 3:**
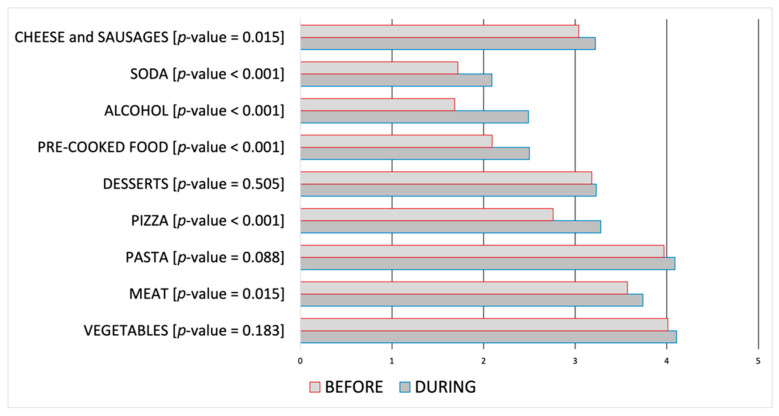
Dietary habits before and during the SARS-CoV-2 lockdown period, within the range 0 (never) to 5 (always).

**Figure 4:**
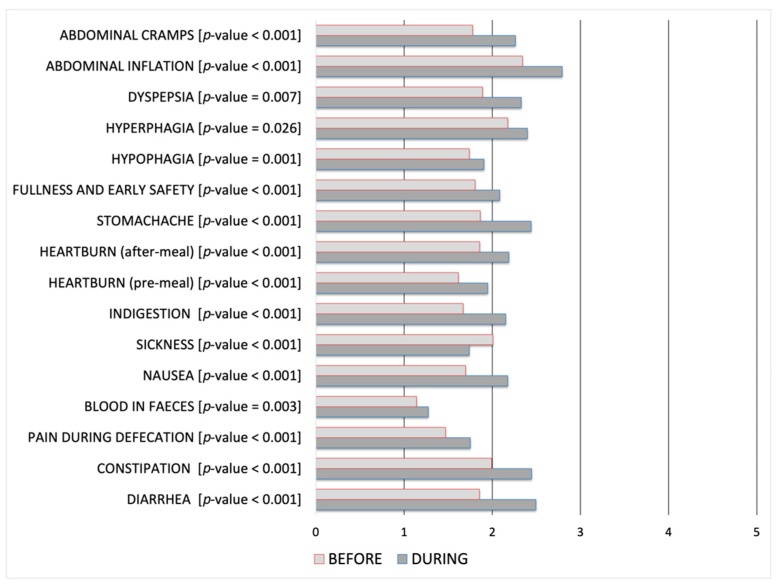
GISs before and during the SARS-CoV-2 lockdown period, within the range 0 (never) to 5 (always).

Furthermore, Figure 3 shows the data related to dietary habits, while the consequent onset of GISs is reported in Figure 4. Statistical significance related to before-during comparison has been reported in Table 7 for Figure 3, and in Table 6 for Figure 4. Data are in Likert-type scale, which represents the related score in a range 0–5 from “Never” to “Always”, an increase in the score represents a worsening; for each alteration, the related *p*-value has also been reported.

Table 8 reports the analysis conducted on anxiety state before and during the lockdown. Anxiety level and anxiety state have been reported above within Table 1, Table 2, Table 3, Table 4 and Table 5.

## 4. Discussion

The COVID-19 pandemic represents the most important public health emergency of the contemporary era. As a consequence of this new global scenario, many people have been suffering from a spike of excruciating psychological issues [22]. The social distancing and quarantine during the COVID-19 pandemic in general and the lockdown period in particular, have limited the activities of the population with increased prevalence of mental disorders [23]. Depression and anxiety are the most common mental illnesses that impact negatively on the quality of life [24]. Several studies indicate a high prevalence of psychological disturbances among medical and non-medical populations [16,17,25]. A study including 1210 respondents from 194 cities in China found that 54% of respondents rated the psychological impact of the COVID-19 outbreak as moderate or severe, 29% reported moderate to severe anxiety symptoms, and 17% reported moderate to severe depressive symptoms [26].

On this basis, the aim of the present study was to investigate self-reported functional GISs of a medical student’s population before and during the first Italian lockdown period and its potential correlation with the anxiety level and dietary habits. In this context, it is important to highlight that Italy was the first European country to announce severe nationwide limits on travel, as the government struggled to stem the spread of a COVID-19 outbreak. In our study, a set of 131 questions was performed on 354 medical students, of which 111 of whom males (31.4%) and 243 females (68.6%). Table S2 (see Supplementary Materials) summarizes demographic data of subjects recruited for this study. Two main reasons can explain the fact that the main part of our cohort is represented by females: (1) in Italy, the majority of medical students are women [27], and (2) females are more active in computer-mediated communication than men [28].

Our statistical analysis showed an anxiety state for 48.9% of interviewed, of which 64.74% report the association with GISs. Medical students show, in general, higher baseline rates of stress and anxiety, compared to the general population [16]. A systematic review of the prevalence of anxiety among this population outside of North America found a large range of prevalence between 7.7% and 65.5% [2]. A more recent meta-analysis analyzes the data from sixty-nine studies comprising 40,348 medical students and found that the global prevalence rate of anxiety among them was 33.8% [15]. Anxiety was most prevalent in the Middle East and Asia subjects, and the subgroup analyses by gender and year of study do not found statistical differences. Moreover, preliminary reports from the literature highlight how anxiety rates remain stable over the pandemic time among medical students or decrease when compared to non-medical ones [29]. This can be explained by a better knowledge of these subjects about incidence, prevalence, diagnosis, and treatments available for SARS-CoV-2 infection versus the non-medical students.

Also, we report statistically significant correlations between GISs before and during the SARS-CoV-2 lockdown for all evaluated symptoms (Table 6). Available literature data indeed note elevated psychiatric symptoms among university students, including anxiety during the lockdown period [30,31]. Our results confirm that stressful life events deeply influence the onset of anxiety in association with GISs. In particular, we found that all the GISs evaluated are statistically significant if compared before and during the lockdown. However, it must be taken into account that a pandemic is an exceptional situation that involves not only a single person or a community, but the worldwide population. Its long time influences and consequences on psychological and functional conditions of the global community are unknown. 

Considered dietary habits, we detected not only the increased consumption of hyper-caloric food as meat, pizza, pre-cooked food, and sweetened drinks but also alcoholic beverages, with a reduction in the intake of fruits and vegetables. Some recent studies carried out in different countries have reported modification in alimentary profiles associated with the lockdown. In particular, increased consumption of foods with high sugar content, such as chocolate and salty snacks, has been reported [32,33]. The possible explanation of these alimentary regimen changes can be explained to the increased levels of anxiety and the difficulties to find open grocery stores close to home [34]. In addition, some evidence from the scientific literature indicates that GISs, and in particular abdominal pain and discomfort, are exacerbated by the intake of specific nourishment as foods rich in carbohydrates, fatty food agents such as alcohol beverages and spices [35]. In this way, we identify in our cohort an unhealthy alimentary pattern profile, that can be associated with the increased perception of GISs. 

Finally, our study was conducted during the critical time of lockdown and social distancing, and to collect the data quickly, we selected a web-based survey method. This solution allows the direct guidance of respondents to a uniform resource locator and the students preferred online surveys [36]. Furthermore, it presents the advantages of saving time in its ease of use with limited cost, the ability to prevent errors, and finally, the rapid transmission of survey results [37]. An online survey may not allow the generalization of results, especially since anxiety and GISs may be due to many other factors other than the pandemic; it is also necessary to consider that the same nature of self-reported data, may result in response biases and in particular for anxiety assessment which may not always be as accurate as being assessed by a mental health professional. However, despite these limitations, our study provides information about the immediate psychological profile of Italian medical students to the COVID-19 pandemic in the gastroenterology setting.

## 5. Conclusions

Our data highlight the pivotal role of the gut-brain axis in human health and its interaction with the alimentary regimen and stressogenic life events to induce GISs. In particular, we highlight the role of stress conditions related to the lockdown, and we found that the concern for one’s health is directly related to a worsening of the GISs in medical students. The data presented show the indirect effects of the COVID-19 pandemic on the gastrointestinal tract and emphasize the role of a prolonged stress condition on psychological health status. Our data provides evidence that a large percentage of medical students have been suffering from anxiety symptoms and changed their dietary habits, during the ongoing pandemic. In this way, a cumulative and critical analysis of the data published in the literature on this topic can be helpful to better define the factors based on GISs and anxiety onset. In the future, the COVID-19 picture may continue to influence people’s lives, and the results of this study may assist in identifying medical students with functional problems, including GISs, so that they can be supported to cognitive-behavioral therapy. 

## Figures and Tables

**Table 1:** Anxiety state: frequency analysis.

Group		Frequency	Percent	Valid Percent	Cumulative Percent
**Before**	NO	331	93.5	93.5	93.5
	YES	23	6.5	6.5	100.0
	Total	354	100.0	100.0	
**During**	NO	181	51.1	51.1	51.1
	YES	173	48.9	48.9	100.0
	Total	354	100.0	100.0	

**Table 2:** Correlation between anxiety state and presence of GISs.

			Presence of GISs
**Spearman’s rho**	**Anxiety state**	**Correlation Coefficient**	0.212
		**Sig. (2-Tailed)**	<0.001
		***n***	354

**Table 3:** Anxiety level before and during the lockdown period.

Group		*n*	Mean	Std. Deviation	Sig. (2-Tailed)
**Before**	Anxiety level	354	13.85	2.997	<0.001
	Valid N (listwise)	354			
**During**	Anxiety level	354	18.12	7.290	
	Valid N (listwise)	354			

**Table 4:** Correlation between anxiety level and presence of GISs during the lockdown: group statistics; see Table 5 for statistical significance.

	GISs	*n*	Mean	Std. Deviation	Std. Error Mean
**Anxiety level**	NO	163	16.08	6.357	0.498
	YES	191	19.85	7.595	0.550

**Table 5:** Correlation between anxiety level and presence of GISs during the lockdown: independent samples.

		Levene’s Test for Equality of Variances		*t*-Test for Equality of Means				
		F	Sig.	t	df	Sig. (2-Tailed)	Mean Difference	Std. Error Difference
**Anxiety level**	Equal variances assumed	6.527	0.011	−5.018	352	<0.001	−3.774	0.752
	Equal variances not assumed			−5.089	351.874	<0.001	−3.774	0.742

**Table 6:** Correlation between GISs before and during the SARS-CoV-2 lockdown.

GISs	*p*-Value
Diarrhea	<0.001
Constipation	<0.001
Pain During Defecation	<0.001
Blood in feces	0.003
Nausea	<0.001
Sickness	<0.001
Indigestion	<0.001
Heartburn (Pre-Meal)	<0.001
Heartburn (After-Meal)	<0.001
Stomachache	<0.001
Fullness and early safety	<0.001
Hypophagia	0.001
Hyperphagia	0.026
Dyspepsia	0.007
Abdominal inflation	<0.001
Abdominal cramps	<0.001

**Table 7:** Correlation between dietary habits before and during the SARS-CoV-2 lockdown period.

Dietary Items	*p*-Value
Fruits/Vegetables	0.183
Meat	0.015
Pasta	0.088
Pizza	<0.001
Desserts	0.505
Pre-Cooked Food	<0.001
Alcohol Beverages	<0.001
Soda	<0.001
Cheese and sausages	0.015

**Table 8:** Correlation between psychometric parameters before and during the SARS-CoV-2 lockdown period.

		*t*	df	Sig. (2-tailed)	Mean Difference	Std. Error Difference	95% Confidence Interval of the Difference	
							Lower	Upper
**Headache**	Equal variances assumed	−9.955	706	<0.001	−0.805	0.081	−0.964	−0.646
	Equal variances not assumed	−9.955	697.555	<0.001	−0.805	0.081	−0.964	−0.646
**Fainting or dizziness**	Equal variances assumed	1.723	706	0.085	0.124	0.072	−0.017	0.266
	Equal variances not assumed	1.723	630.337	0.085	0.124	0.072	−0.017	0.266
**Chest pain or discomfort**	Equal variances assumed	<0.001	706	1.000	<0.001	0.074	−0.145	0.145
	Equal variances not assumed	<0.001	647.664	1.000	<0.001	0.074	−0.145	0.145
**Back pain**	Equal variances assumed	−8.094	706	<0.001	−0.686	0.085	−0.853	-0.520
	Equal variances not assumed	−8.094	704.141	<0.001	−0.686	0.085	−0.853	−0.520
**Stomach pain and nausea**	Equal variances assumed	−5.172	706	<0.001	−0.421	0.081	−0.581	−0.261
	Equal variances not assumed	−5.172	705.819	<0.001	−0.421	0.081	-0.581	−0.261
**Muscle aches**	Equal variances assumed	−14.273	706	<0.001	−0.975	0.068	−1.109	−0.841
	Equal variances not assumed	−14.273	672.848	<0.001	−0.975	0.068	−1.109	−0.841
**Shortness of breath**	Equal variances assumed	−8.587	706	<0.001	−0.556	0.065	−0.684	−0.429
	Equal variances not assumed	−8.587	701.109	<0.001	−0.556	0.065	−0.684	−0.429
**Hot/Cold flash**	Equal variances assumed	−8.408	706	<0.001	−0.551	0.066	−0.679	−0.422
	Equal variances not assumed	−8.408	701.436	<0.001	−0.551	0.066	−0.679	−0.422
**Lump in the throat**	Equal variances assumed	−8.393	706	<0.001	−0.554	0.066	−0.683	−0.424
	Equal variances not assumed	−8.393	695.004	<0.001	−0.554	0.066	−0.683	−0.424
**Weakness**	Equal variances assumed	−11.863	706	<0.001	−0.799	0.067	−0.932	−0.667
	Equal variances not assumed	−11.863	674.446	<0.001	−0.799	0.067	−0.932	−0.667
**Feeling that your arms or legs are heavy**	Equal variances assumed	−9.521	706	<0.001	−0.661	0.069	-0.797	-0.525
	Equal variances not assumed	−9.521	681.280	<0.001	−0.661	0.069	−0.797	−0.525
**Abnormal sensations of numbness and tingling**	Equal variances assumed	−19.020	706	<0.001	−1.130	0.059	−1.247	−1.013
	Equal variances not assumed	−19.020	519.731	<0.001	−1.130	0.059	−1.247	−1.013

## Data Availability

The data presented in this study are available on request from the corresponding author. The data are not publicly available due to privacy limitation.

## Supplementary Materials

The following are available online at https://www.mdpi.com/2077-0383/10/6/1221/s1, Table S1: The survey consists of the following main categories: socio-demographic characteristics, GISs and psychometric parameters, Table S2: Descriptive characteristics of the respondent cohort.
