# Supplementary material for: Anxiety and Gastrointestinal Symptoms Related to COVID-19 during Italian Lockdown
Source: J Clin Med. 2021 Mar 16;10(6):1221. doi: 10.3390/jcm10061221 (PMC8001711; doi:10.3390/jcm10061221)
Supplement: Supplementary file 1 [file jcm-10-01221-s001.pdf]

Supplementary Material

Anxiety and Gastrointestinal Symptoms related to COVID-19 during Italian Lockdown

Ludovico Abenavoli [1,*], Pietro Cinaglia [2], Giuditta Lombardo [3], Eduardo Boffoli [4], Miriam Scida [4], Anna Caterina Procopio [1], Tiziana Larussa [1], Luigi Boccuto [5], Christian Zanza [6,7]; Yaroslava Longhitano [7], Sharmila Fagoonee [8], and Francesco Luzza [1]

[1] Department of Health Sciences, University "Magna Graecia" Catanzaro, Italy; l.abenavoli@unicz.it (L.A.), procopioannacaterina@gmail.com (A.C.P.), tiziana.larussa@gmail.com (T.L.), luzza@unicz.it (F.L.)
[2] Department of Surgical and Medical Sciences, University "Magna Graecia", Catanzaro, Italy; cinaglia@unicz.it (P.C.)
[3] Psychologist, Independent Researcher, Catanzaro, Italy; giuditta.lombardo@libero.it (G.L.)
[4] School of Medicine, University "Magna Graecia", Catanzaro, Italy; eduardo.boffoli96@gmail.com (E.B.), scidamiriam0@gmail.com (S.M.)
[5] School of Nursing, College of Behavioral, Social and Health Sciences, Clemson University, Clemson, SC, USA; lboccut@clemson.edu (L.B.)
[6] Department of Anesthesia, Critical Care Medicine and Emergency Medicine, Michele and Pietro Ferrero Hospital, Verduno; Italy; christian.zanza@live.it (C.Z.)
[7] Department of Anesthesia and Critical Care Medicine, AON SS Antonio e Biagio e Cesare Arrigo, Alessandria, Italy; lon.yaro@gmail.com (Y.L.)
[8] Institute of Biostructure and Bioimaging, National Research Council, Molecular Biotechnology Center, Turin, Italy; sharmila.fagoonee@unito.it (S.F.)

* Correspondence: l.abenavoli@unicz.it; Tel.: +39-0961-3694387

Table S1. The survey consists of the following main categories: socio-demographic characteristics, GISs and psychometric parameters.

Category	Question	Answer
Socio-demographic characteristics	Sex	Male, Female
	Age	Free field
	Nationality	Free field
	Marital status	Single, Dating, Married, Divorced
	Educational level	Undergraduate, Graduate
	Region of residence	List of Italian Regions
	Do you live in your Region of residence?	Yes, No
	Where are you live actually?	List of Italian Regions
GISs: history	Have you ever suffered from any of these ailments? Diarrhea, constipation, pain during defecation, blood in feces, nausea, vomit, indigestion, heartburn (pre-meal), heartburn (after-meal), stomachache, fullness and early safety, hypophagia, hyperphagia, dyspepsia, abdominal inflation, abdominal cramps.	Yes, No for each symptom in the list.
	Positive family history for a GISs.	Yes, No

GISs<br><br>*BEFORE and DURING lockdown*	Diarrhea, constipation, pain during defecation, blood in feces, nausea, vomit, indigestion, heartburn (pre-meal), heartburn (after-meal), stomachache, fullness and early safety, hypophagia, hyperphagia, dyspepsia, abdominal inflation, abdominal cramps.	Multiple-choice (Never, Rarely, Sometimes, Often, and Always) for each symptom in the list.
Dietary habits<br><br>*BEFORE and DURING lockdown*	Food consumption of fruits/vegetables, meat, pasta, pizza, desserts, pre-cooked food, alcohol, soda, cheese, and sausages.	Multiple-choice (Never, Rarely, Sometimes, Often, and Always) for each food in the list.
Psychometric evaluation: anxiety level<br><br>*BEFORE and DURING lockdown*	Headache	Multiple-choice (Never, Rarely, Sometimes, and Often) for each question.
	Fainting or dizziness	
	Chest pain or discomfort	
	Back pain	
	Stomach pain and nausea	
	Muscle aches	
	Shortness of breath	
	Hot/Cold flash	
	Lump in the throat	
	Weakness	
	Feeling that your arms or legs are heavy	
	Abnormal sensations of numbness and tingling	
Psychometric evaluation: SHAI test<br><br>*BEFORE and DURING lockdown*	I am concerned with health	Multiple-choice (Never, Rarely, Sometimes, and Often) for each question.
	I pay attention to the pains	
	I notice sensations or changes in my body	
	I can keep myself from thinking about diseases	
	I am afraid of having a serious disease	
	I have mental images of myself sick	
	I'm having trouble not thinking about my health	
	If the doctor tells me I have nothing how much I keep thinking about it	
	If I hear about a disease, I think I may have it too	
	If I feel a physical sensation or a change, I wonder what it means	
	It is possible that I am ill with something serious	
	I always think I'm seriously sick	
	If I notice a bodily sensation that I can't explain, how much I think about it?	
	My friends and family would say that I care about my health	

Table S2. Descriptive characteristics of the respondent cohort.

**Mean age (SD)**	23.93 (3.67)	
**Sex**	Male	31.4%
	Female	68.6%
**Nationality**	Italian	99.2%
	Polish	0.6%
	Croatian	0.3%
**Education level**	Undergraduate	72.3%
	Graduate	27.7%
**Marital status**	Single	48.0%
	Dating	49.2%

	Married	2.5%
	Divorced	0.3%
	Unknown	0.3
**Region of residence**	Abruzzo	1.1
	Basilicata	1.4
	Calabria	48.0
	Campania	3.1
	Emilia	2.3
	Friuli	8.2
	Lazio	3.1
	Liguria	0.8
	Lombardy	4.8
	Marche	7.1
	Piedmont	1.1
	Apulia	5.4
	Sardinia	0.3
	Sicily	3.7
	Tuscany	1.7
	Trentino	0.6
	Veneto	7.1
**Status for the region where the subject lives**	Resident	90.7%
	Non-resident	9.3%
